# Clinicopathological and immunohistochemical analysis of spindle cell carcinoma of the larynx or hypopharynx: A report of three cases

**DOI:** 10.3892/ol.2014.2172

**Published:** 2014-05-23

**Authors:** YI ZHENG, MANG XIAO, JIANGUO TANG

**Affiliations:** Department of Otolaryngology-Head and Neck Surgery, Sir Run Run Shaw Hospital Medical College of Zhejiang University, Hangzhou, Zhejiang 310016, P.R. China

**Keywords:** spindle cell, squamous epithelial, larynx, hypopharynx, immunohistochemistry

## Abstract

Spindle cell carcinoma (SpCC) is a rare and unusual biphasic malignant tumor, which involves sarcomatoid proliferation of pleomorphic spindle cells and squamous cell carcinoma. There are various reports on the clinical and pathological findings of SpCC in the head and neck; however, this type of tumor remains uncommon in the larynx and hypopharynx. The histogenesis of SpCC has been the subject of debate for many decades. Although it is generally accepted that SpCC is a monoclonal epithelial neoplasm, and the spindle cell element is derived from squamous epithelium with divergent mesenchymal differentiation, this type of tumor poses a significant diagnostic challenge to pathologists and clinicians with regard to the therapeutic approach. In the present report, three cases of SpCC of the larynx or hypopharynx were investigated. The histological and immunohistochemistry findings are presented to provide further data on this rare type of tumor.

## Introduction

According to previous studies, spindle cell carcinoma (SpCC) is an unusual form of divergent differentiated squamous cell carcinoma (SCC), which consists of elongated (spindle) epithelial cells and resembles a sarcoma (1). SCC, with the spindle cell component, is an uncommon phenomenon and a rare type of malignant tumor. It is also termed as a sarcomatoid carcinoma, pseudosarcoma, pleomorphic carcinoma and sarcomatous carcinoma (2,3). There are numerous reports describing the clinical and pathological findings of SpCC in the head and neck (4–7), with the majority described as being located in the oral cavity, larynx, tonsils and pharynx. However, SpCC occurs elsewhere in the body, such as the skin, lungs and breasts, and the symptoms vary according to the site.

The histogenesis of SpCC has been the subject of debate for many decades. It is generally accepted that SpCC is a monoclonal epithelial neoplasm (8–11) and the spindle cell element is derived from squamous epithelium with divergent mesenchymal differentiation (5). However, this type of tumor poses a significant diagnostic challenge to the pathologist due to the remarkable morphological and immunohistochemical overlap with other benign and malignant spindle cell tumors (2,12). Therefore the importance of an accurate diagnosis is emphasized in view of the different therapeutic approaches that are required.

In the present report, three cases of SpCC of the larynx or hypopharynx were investigated, with the aim of presenting further data on the clinicopathology and immunohistochemistry of this rare type of tumor. Patients provided written informed consent.

## Case report

### Case one

**Figure 1:**
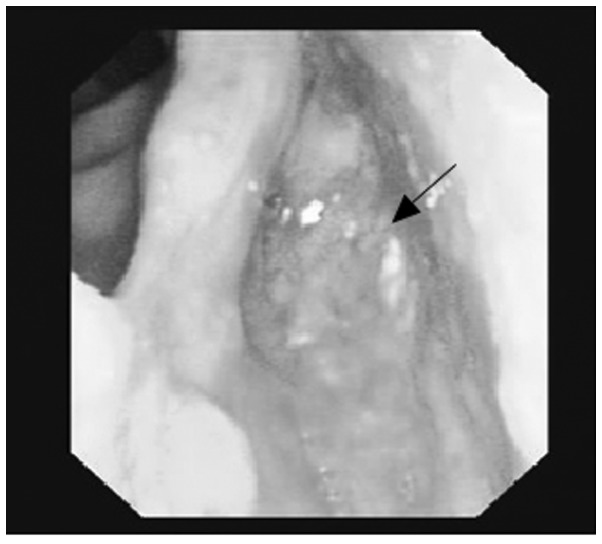
Endoscopy of case one revealed a 1.5-cm submucosal mass in the left pyriform sinus, which extended to the lateral wall.

**Figure 2:**
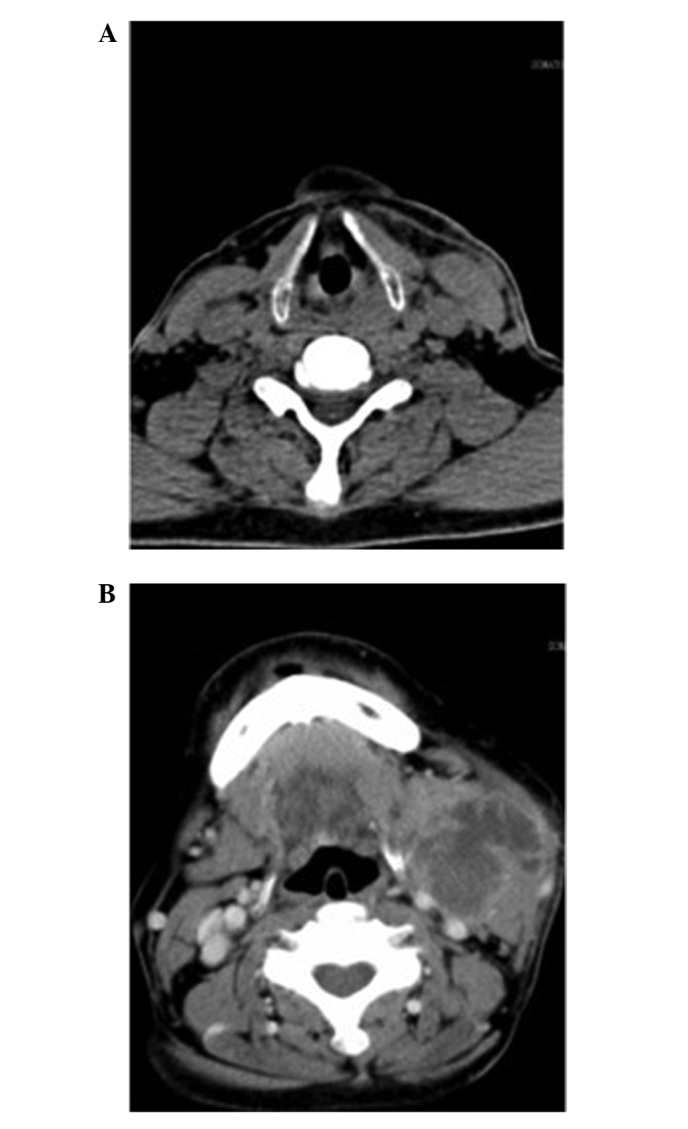
Computed tomography findings of case one. (A) A soft tissue mass was demonstrated in the left pyriform sinus. (B) A 4.4×4.1-cm lesion, which was not well defined from the surrounding healthy soft tissue, was demonstrated on the left side of the neck.

A 55-year-old male was admitted to the Department of Otolaryngology and Head and Neck Surgery, Sir Run Run Shaw Hospital, Medical College of Zhejiang University (Hangzhou, China) complaining of a mass on the left side of the neck, which had been present for six months. The patient reported that the mass had increased rapidly over the two preceding months. The patient stated there was no tenderness or paresthesia, however, the mass had been punctured and pus had been extracted at the Jiangshan Beilin Hospital (Jiangshan, China). An endoscopy revealed a 1.5-cm submucosal mass in the left pyriform sinus, which extended to the lateral wall (Fig. 1). The posterior pharyngeal wall, vocal cords, subglottic region and the base of the tongue appeared to be healthy. Computed tomographic (CT) examinations demonstrated a soft tissue mass in the left pyriform sinus and a 4.4×4.1-cm lesion, which was not well defined from the surrounding healthy soft tissue on the left side of the neck (Fig. 2).

The patient underwent surgical removal of the mass in the left pyriform sinus, without involvement of the larynx, followed by radial forearm free flap (RFFF) reconstruction of the hypopharynx under general anesthesia. A neck dissection was performed to treat the neck lymph node metastasis.

Following surgery, the patient underwent chemoradiation therapy; this consisted of radiotherapy (6 MV single-wavelength anomalous diffraction X-ray; absorbed dose to the tumor was 3,600 cGy; 18 fractions for 26 days) plus concurrent chemotherapy of 170 mg oxaliplatin for one day and 140 mg nedaplatin for one day. No acute side-effects were noted, however, mucositis and odynophagia were observed. Following discharge from hospital the patient was administered with Chinese traditional medicine, including *Angelica*, *Astragalus*, *Prunella* and toad skin. During the follow-up examination 8 months following the patient’s surgery no evidence of recurrence or metastasis was identified.

### Case two

**Figure 3:**
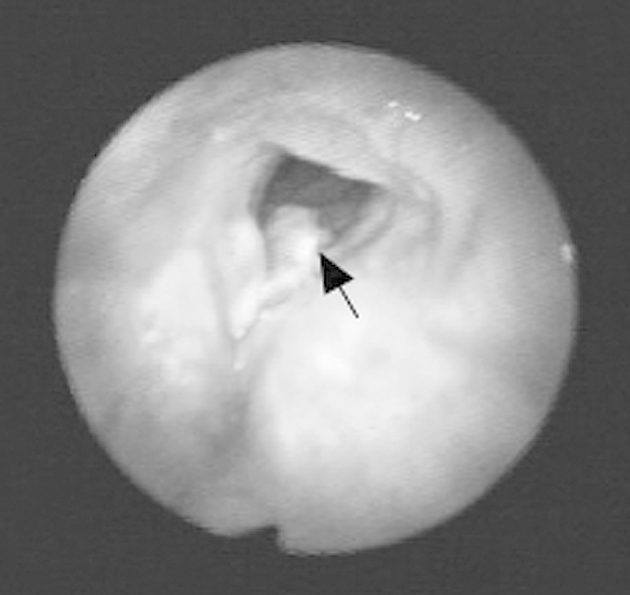
Endoscopy of case two demonstrated a mass on the left vocal cord, which markedly extended to the anterior commissure.

**Figure 4:**
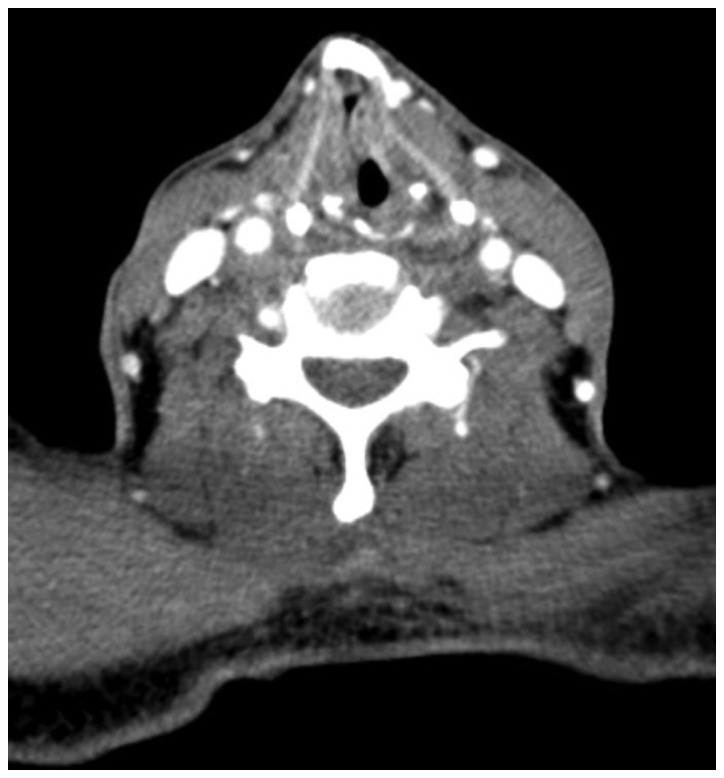
Computed tomography findings of case two showed a 1.5×1.0-cm mass on the left vocal cord.

A 62-year-old male was admitted to the Department of Otolaryngology and Head and Neck Surgery, Sir Run Run Shaw Hospital (Hangzhou, China), to evaluate the presence of persistent hoarseness (six-month duration). An endoscopy demonstrated a mass on the left vocal cord, which markedly extended to the anterior commissure (Fig. 3). CT showed a 1.5×1.0-cm mass on the left vocal cord, at high resolution (Fig. 4). A total laryngectomy with neck dissection was performed. The surgically-removed tumor of the left vocal cord appeared cauliflower-like and was 1.5×1.3 cm in size. Follow-up of the patient 6 months postoperatively revealed pulmonary metastases.

### Case three

**Figure 5:**
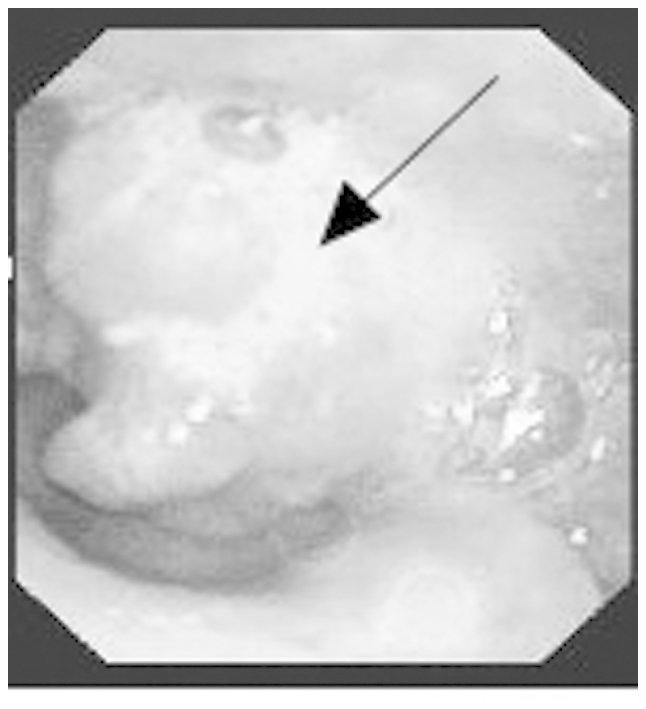
Endoscopy of case three demonstrated the mass on the posterior wall of the hypopharynx.

**Figure 6:**
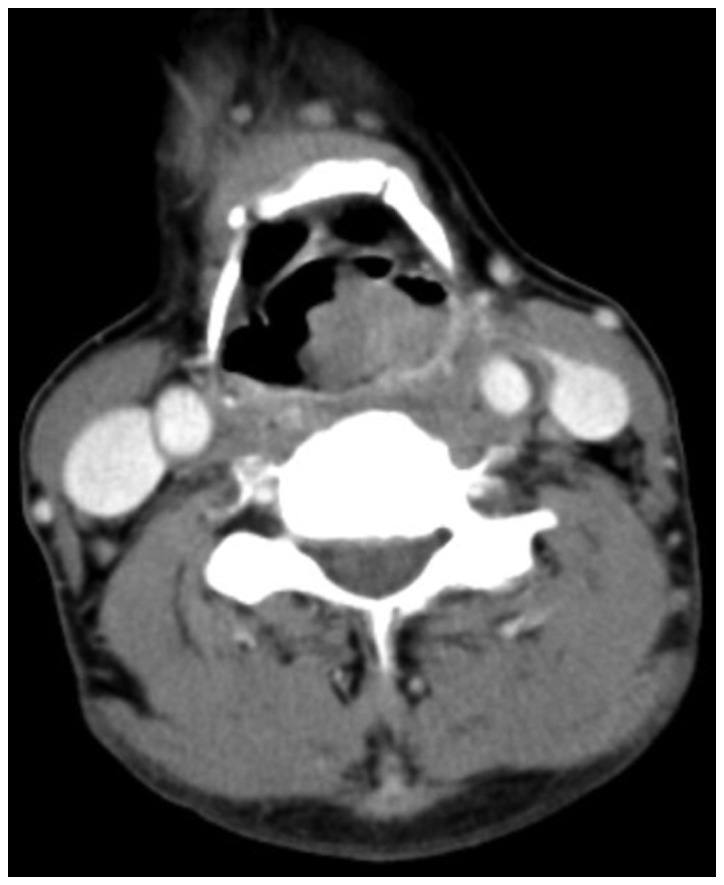
Computed tomography findings of case three showed a 3.3×4.0-cm mass on the posterior wall of the hypopharynx.

A 57-year-old male presented at the Department of Otolaryngology and Head and Neck Surgery, Sir Run Run Shaw Hospital (Hangzhou, China) with a one-year history of pharyngeal foreign body sensation. An endoscopy and CT revealed a large mass on the posterior wall of the hypopharynx (Figs. 5 and 6). The patient was treated with a near total hypopharyngectomy followed by RFFF reconstruction. In addition, external radiotherapy was administered at the Taizhou Hospital (Taizhou, China). Metastases and recurrence were not clinically apparent at the 5.5-month follow-up. A summary of all three cases is presented in Table I.

### Histopathological and immunohistochemical findings

**Figure 7:**
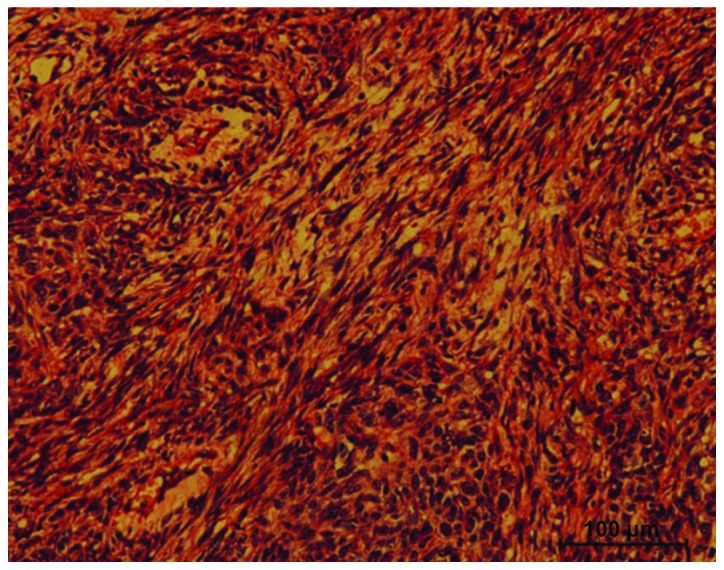
Bundles of spindle cells exhibiting pleomorphism, hyperchromatism and abnormal mitoses (hematoxylin and eosin stain; magnification, ×20).

The immunohistochemical results of SpCC are demonstrated in Table II. In case one, macroscopically, the largest mass of the neck was 6×5.5 cm, smooth and solid with partly cystic degeneration, while the tumor of the left pyriform sinus was 1.5×1.2×1.7 cm and exhibited surface ulcers. The tissue sample from case two was the total larynx, with a 1.5×1.3-cm cauliflower-like mass obscuring the entire left vocal cord. In case three, the mass of the posterior wall of the hypopharynx was also cauliflower-like, ~6.5×6 cm in size and extended to the left pyriform sinus. Histologically, the tumors all demonstrated a biphasic appearance. The tumors were composed of bundles of spindle cells with an unusual, basophilic, hyperchromatic, pleomorphic appearance accompanying small areas of SCC (Fig. 7). In addition, various quantities of collagen were identified in the sarcomatoid zones.

**Figure 8:**
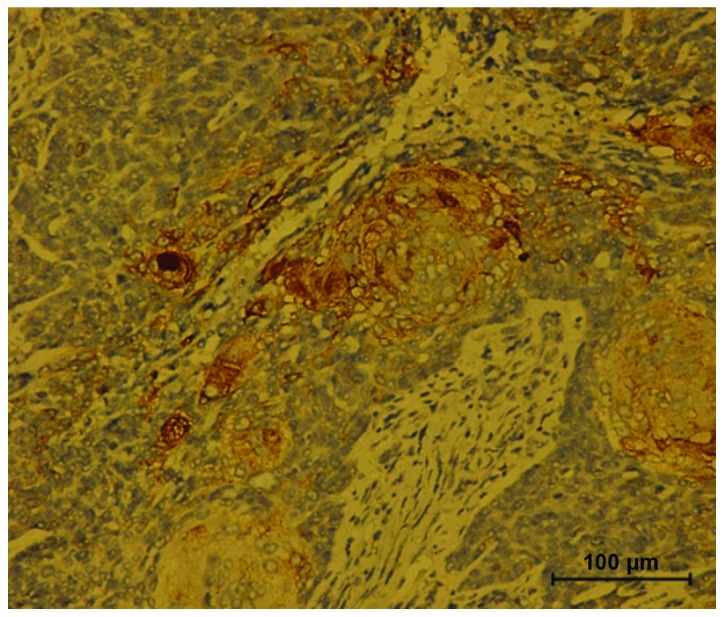
Spindle cells demonstrating positive expression for epithelial membrane antigen using (diaminobenzidine stain; magnification, ×20).

**Figure 9:**
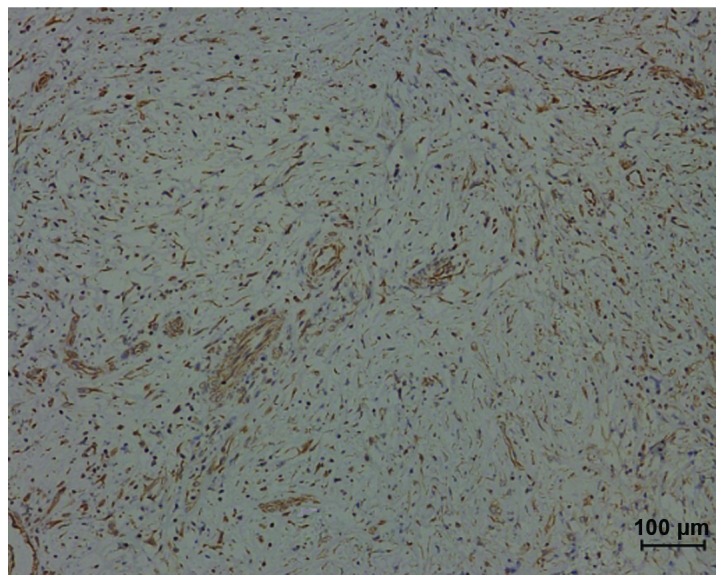
Spindle cells demonstrating positive expression for vimentin using (streptavidin-peroxidase stain; magnification, ×10).

Immunohistochemistry revealed that the SCC component was strongly positive for cytokeratin (CK) and the spindle cell component was strongly positive for vimentin (VM; Fig. 9). Reactivity for epithelial membrane antigen (Fig. 8), Ki-67, smooth muscle actin and actin were detected at various levels. No immune activity was observed for desmin, CD34 or CK7. Additional immunohistochemical data is demonstrated in Table II. Case one (with lymph node metastasis) exhibited well-differentiated SCC on the left side of the neck, however, there was no evidence of metastases in the other two cases.

## Discussion

SpCC is a rare and unusual biphasic malignant neoplasm of the head and neck. It consists of sarcomatoid proliferation of pleomorphic spindle shape cells and SCC (13). The mean age of diagnosis of SpCC is 57 years (14). Four factors were considered that may predispose individuals to this disease: i) Tobacco use; ii) alcohol use; iii) poor oral health; and iv) previous irradiation at the site of the tumor (8). The patients included in the present study were aged 55–62 years and had significant histories of smoking and alcohol consumption.

SpCC develops due to a variety of reasons, including genetic predisposition, however, it may also be caused by a combination of other factors, including injury and inflammation in patients that are thought to be predisposed to this type of tumor. It has been hypothesized that the development of the spindle cell phenotype involves a functional loss of genes, which control epithelial differentiation, and that the conversion to the spindle morphology is a recessive entry (15). Lane (16) and Battifora (17) regarded the spindle cells as varying between mesenchymal metaplasia of epithelial cells to an atypical, although benign, stromal response. Although there is disagreement with respect to the origin of these elements, there is a consensus among various individuals that the size, location and presence of neck disease, and not the history *per se*, may guide the selection of the therapeutic options and influence patient survival. One study has noted that the malignant squamous cell component may be inconspicuous, and thus a diligent search for these elements is required in order to obtain an accurate diagnosis (18).

Histopathologically, the microscopic features of SpCC include the presence of two distinct epithelial-derived components; a squamous cell and a sarcomatoid spindle cell component. The squamous cell component forms a minor portion of the tumor mass, whereas the spindle cell component constitutes the greatest portion and presents a wispy and fasciculated pattern, which was also demonstrated in the present cases. The squamous component may be represented by dysplasia, carcinoma *in situ* or frankly invasive carcinoma (19). The patient in case one exhibited SCC *in situ*.

Histological studies alone cannot explain the spindle cell components. Recent immunohistochemical studies have demonstrated the histogenesis of the spindle cells within these tumors. The concept that spindle cell elements are epithelial in origin is currently verified by positive keratin immunostaining; in addition, the demonstration of desmosomes and tonofilaments in the cells provides further support (7,20). CK is considered to be the most sensitive and reliable epithelial marker used for demonstrating the epithelial phenotype. In the present study, the spindle cells were positive for VM and negative for CK. The VM positivity indicated that these bundles of cells are carcinoma cells with true mesenchymal metaplasia. By employing staining for ras oncogene p21, CK and VM, Toda *et al* (21) proposed that the spindle cell component is epithelial in origin and is malignant. As a result of the present study, it is hypothesized that SpCC are of epithelial origin, however, undergo an alteration that results in a loss of CK.

Review of the literature, diagnostic imaging, specific staining and electron microscopy facilitates with the categorization of this type of tumor and therefore, in the design of individualized surgical approaches. Batsakis (14) advises that these lesions be viewed as aggressive and that therapy should be based on clinical staging rather than microscopy. Diagnostic imaging may aid with delineating the extent of this type of lesion. In the present study, CT was required to perform the surgical procedures on the patients. Surgical removal is the preferred method, with radiation providing an effective adjunctive therapy. Certain authors are of the opinion that surgery alone is not sufficient and that radiotherapy should be a mandatory adjunct to surgery. Radiotherapy is also significant in cases where the surgical margin is positive or where there is extensive nodal disease. In a previous study, the overall recurrence rate of SpCC of the head and neck was identified as 71.4% with a metastasis rate of 21.4% (22). These metastasis locations may be nearby tissues or system-wide locations, which include the lungs, kidneys and the liver. Incidentally, the lungs were the most frequent site for metastasis, as reported by Thompson *et al* (2) who did not identify soft tissue as a site for metastasis in their large population of patients. In those particular cases, the prognosis was poor and chemotherapy and radiation were the only methods for controlling the cancer.

In conclusion, SpCC of the larynx and hypopharynx is potentially aggressive, appears to readily recur and metastasize, and patients generally have a poor prognosis. Batsakis (14) identified an overall mortality rate of 35% within 2.5 years across all anatomical sites of the head and neck that may be associated with this type of tumor. Distant metastases and the depth of tumor invasion into underlying structures were found to be reliable prognostic factors, together with their polypoid configuration. Therefore, long-term and frequent follow-up is considered to be essential.

## Figures and Tables

**Table I:** Brief summary of the three cases.

No.	Gender	Age, years	Symptom	Site of tumor	Size, cm	Neck lymph node metastasis	Follow-up
1	Male	55	Neck mass	Left pyriform sinus	1.5×1.2	1	No recurrence at 8 months
2	Male	62	Hoarseness	Left vocal cord	1.5×1.3	0	Pulmonary metastases at 6 months
3	Male	57	Foreign body sensation	Posterior wall of the hypopharynx	6.5×6.0	0	No recurrence at 5.5 months

**Table II:** Immunohistochemical results of the spindle cell carcinoma.

No.	CK (high)	CK (low)	p63	CK	EMA	Desmin	VM	Ki-67	CD34	SMA	Actin	CK7
1	+	−	+	+	−	−	+	+	−	−	−	/
2	+	/	/	+	−	−	+	+	−	+	+	−
3	+	+	+	+	+	/	+	−	/	+	/	−

CK, cytokeratin; EMA, epithelial membrane antigen; VM, vimmentin; CD, cell adhesion; SMA, smooth muscle actin; +, positive; −, negative; /, not conducted.
